# Agricultural drought in the Vietnamese Central Highlands at 1-km resolution: Monthly and annual datasets

**DOI:** 10.1016/j.dib.2023.109194

**Published:** 2023-05-02

**Authors:** Thuong V. Tran, Duy X. Tran, Duy B. Nguyen

**Affiliations:** a Institute of Engineering and Technology, Thu Dau Mot University, 6 Tran Van On, Thu Dau Mot city, Binh Duong 75000, Viet Nam; b Grasslands Research Centre, AgResearch Ltd, Tennent Drive, 11 Dairy Farm Road, Palmerston North 4442, New Zealand; c Department of Photogrammetry and Remote Sensing, Hanoi University of Mining and Geology, 18 Pho Vien, Bac Tu Liem, Hanoi 10000, Vietnam

**Keywords:** Drought index, iDMI, TCI, VCI, ESI, Tay Nguyen

## Abstract

Drought is a complex natural hazard which can create significant impacts on society and environment. Given that this phenomenon varies across space and changes over time dependent on various factors (e.g., physical conditions and human activities), the available of spatiotemporal drought data enables a better monitoring and assessment of drought severity This study introduced the integrated multivariate drought index (iMDI) data, a new regional drought index, at 1 km spatial and monthly temporal resolutions for the Vietnamese Central Highlands over a 20-years period. The iMDI was developed recently which is a combination of vegetation condition index (VCI), the temperature condition index (TCI), and the evaporative stress index (ESI) based on the feature of scaling algorithms (i.e., normalisations and standardisation). The data were processed using the median values of MODIS time-series imagery obtained from the Google Earth Engine (GEE) platform. The iMDI datasets are available for monthly and annual drought monitoring between 2001 and 2020. Additionally, the datasets of VCI, TCI, and ESI were provided so that users can apply for their own purposes even though these data can directly obtain from GEE or other sources. Users, especially those without technical expertise, can reap the advantages of having open access to iDMI data. By doing so, they can reduce their expenses and the time required to process data. As such, this accessibility can promote the use of data for diverse applications, such as evaluating the impact of droughts on the environment and human activities and monitoring droughts regionally.

**Specifications Table**

**Table:** 

Subject	Environmental Sciences
Specific subject area	Remote sensing for drought monitoring
Type of data	Georeferencing Tagged Image Format - GeoTIFF (*.tif)
How the data were acquired	The new regional drought index, namely integrated Multivariate Drought Index (iMDI) [1], was generated based on the combination of the vegetation condition index (VCI) [2], the temperature condition index (TCI) [2], and the evaporative stress index (ESI) [3]. Monthly and annual time series data were created during the 2001–2020 period for agricultural drought monitoring and assessment. The gridded datasets were generated at 1 km × 1 km in the Vietnamese Central Highlands.
Data format	Raw (Georeferenced raster dataset)
Description of data collection	The iMDI synthesized the moisture deficits, soil thermal stress, and vegetation growth status in drought processes and makes it favorable for the comprehensive agricultural drought monitoring. The combination of three proposed indices (i.e., VCI, TCI, and ESI) provides more accurate and comprehensive information on drought severity in connection to local conditions. The feasibility of the iMDI is also proved in the correlation with ground-based drought data in the study area. At present, the products include monthly and annual per-pixel drought with 1 km spatial resolution, covering the period of 2001 -2020.
Data source location	City/Town/Region: Central Highlands Country: Viet Nam Latitude and longitude: 11°12”00”–15°27”15” N and 107°12–108°59’37” E All images are obtained from the satellite Moderate Resolution Imaging Spectroradiometer (MODIS) based on GEE platform.
Data accessibility	Repository name: Mendeley Data Data identification number: http://doi.org/10.17632/w2dzy4r6vb.3 Direct URL to data: https://data.mendeley.com/datasets/w2dzy4r6vb/3
Related research article	Tran, T. V., Bruce, D., Huang, C. Y., Tran, D. X., Myint, S. W., & Nguyen, D. B. (2023). Decadal assessment of agricultural drought in the context of land use land cover change using MODIS multivariate spectral index time-series data. *GIScience & Remote Sensing, 60*(1), 2163070 https://doi.org/10.1080/15481603.2022.2163070

## Value of the Data

• Most drought information available are single drought index or point-based data collected at the weather stations, yet multivariate and spatially-explicit regional drought data is still limited. The iMDI product provides valuable temporal (monthly and annual) and per-pixel (1-km resolution) agricultural drought data.
• The iMDI data has a potential respond to moisture content in vegetation biomass associated with water stress which directly related to agricultural droughts. This data enables a wide range of regional drought information to be examined such as drought severity, pattern, duration, trend, and spatial extent.
• The development of multivariate drought index often requires a substantial amount of time and expense. The available of processed drought index is valuable, as this free of charge and readily used data will promote the use of drought data in practices, especially useful for non-technical people who have limited skills in remote sensing data processing.
• These data are particularly useful for environmental scientists who are interested in using drought datasets to identify trends and patterns in drought occurrence and understand of how drought conditions are changing across space. Besides, information obtained from the drought datasets in addition to socio-economic and environmental data can be used by policymakers and land and environmental planners to develop spatial-based (i.e., place-based) drought mitigation strategies.
• Given the increasing drought event and magnitude due to climate change, the drought data from this study can be used to identify drought risks and optimise land use land and land cover pattern and agricultural land management practices to adapt and mitigate to the current and future effects of climate change.

## 1 Objective

Drought monitoring is an important aspect of resource planning and management as it helps identify areas that are severely impacted by or vulnerable to drought risks over time [4,5]. As such, the availability of spatiotemporal drought data is essential. Understanding drought severity and its pattern is important to assess the impacts of drought conditions on agricultural production, ecosystem health, and people well-being [6,7]. Although several types of drought data have been produced and made available online, most of current regional and global drought data are developed based on single drought index. The availability of drought data that takes into account various environmental factors such as precipitation, soil moisture, and evapotranspiration is still limited. Therefore, the objective of this this study is to provide a well-described monthly and annual dataset of agricultural drought (i.e., the integrated Multivariate Drought Index - iMDI) using the method proposed by Tran et al. [1]. The data are available in raw of georeferenced raster formats (GeoTIFF), so that researchers can reproduce or reuse the results for their own purposes in the field of drought monitoring under various GIS software and platforms. Data input used for iMDI computation (i.e., TCI, VCI, and ESI) was also provided. This addition enables users to be more flexible in using data for their practices..

## 2 Study Area

**Fig. 1:**
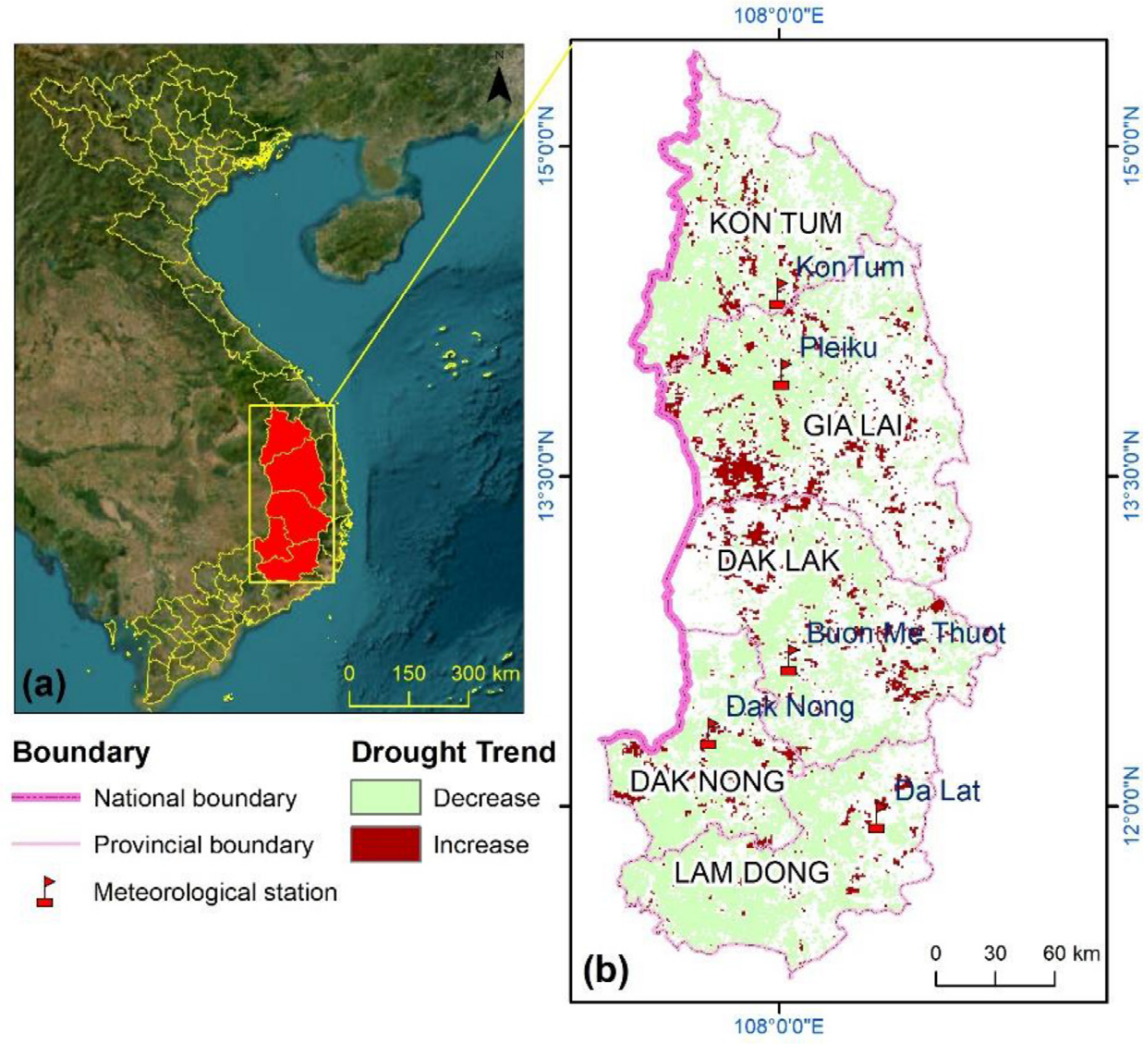
(a) Location of the study area and, with (b) meteorological stations, and annual drought trend. The trend was derived from the annual iMDI during the 2001–2020 period, based on the Ordinary Least Square algorithm.

The Vietnamese Central Highlands is located from 11°12′00” to 15°27′15” north latitude and from 107°12 to 108°59′37” east longitude (Fig. 1). The region includes five administrative provinces: Kon Tum, Gia Lai, Dak Lak, Dak Nong, and Lam Dong, with a total area of 54,474 km^2^. The study area is listed as one of the specialised areas in perennial commercial crops in Vietnam, focuses primarily on coffee cultivation, accounting for 85% of the cultivated land and 90% of the production in the country [8]. However, the exacerbation of drought severity in relation to deforestation has been taken place that created severe influences on water supply for perennial crops (mainly coffee) and a part of rice and cash crops in recent decades [9,10]. In the dry season of 2015–2016 period, the Vietnamese government provided 45 million USD worth of relief and disaster support services for drought-affected to the Central Highlands [9,11]. The limited number of meteorological stations - with each province only having one - presents a challenge for monitoring drought in the Highlands. To address this challenge, there is a growing need for large-scale, long-term, and cost-effective monitoring and mapping techniques that utilise readily available remotely sensed data. This will enable the provision of practical information to support local governments in developing mitigation strategies for the impacts of drought.

## 3 Data Description

**Fig. 2:**
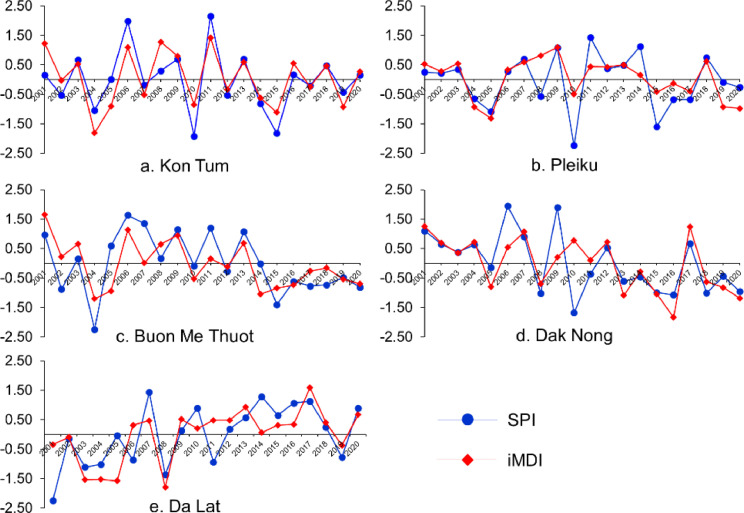
The temporal variation of annual SPI and iMDI in the 2001 – 2020 period.

The dataset includes monthly and annual iMDI at a 1 km spatial resolution for the Vietnamese Central Highlands. Also, the vegetation condition index (VCI), the temperature condition index (TCI), and the evaporative stress index (ESI) are involved. The data was compared with drought events based on the datasets of the real drought events that occurred and reported in the historical period of the region. In addition, the drought variations in association with land use land cover patterns were analysed in the study area to further investigate the feasibility of the iMDI. Fig. 1 shows the location and trend in drought pattern of the study area from 2001 to 2020. Fig. 2 presents the annual patterns of iMDI and ground-based Standardised Precipitation Index (SPI) from 2001 to 2020.

The drought conditions in the study area was categorized into five categories in order to offer a uniform interpretation of the drought assessment (Table 1). The datasets are projected in the zone 48 North Universal Transverse Mercator (UTM) coordinate system, using the ED50 ellipsoid. There is individual file for each dataset, and for each drought index and time scale. The files are formatted under raster files and available in Georeferencing Tagged Image Format (.tif) from 2001 to 2020. All data are stored in Mendeley cloud-based communal repository data and can be accessed freely at https://data.mendeley.com/datasets/w2dzy4r6vb/3. There are two types of data, including raw files and legend files, that users can use for their studies. The raw data showed the original values for each pixel, which can be used with different models or algorithms. The legend files showed the different types of drought classification that can be used for attribute analysis: 1-extreme drought, 2-severe drought, 3-moderate drought, 4-near drought, and 5-no drought (Table 1). Also, the VCI, TCI, and ESI datasets are included so that users can use them for their own needs, even though these datasets can be obtained directly from Google Earth Engine (GEE) or other sources. Each folder includes three sub-folders: iDMI, VCI, TCI, and ESI. Note that our study mainly provided the iMDI, while the rest of drought indices only support for the practical process.

**Table 1:** The drought categories based on iMDI dynamic range and relative categories [1].

Category	Code	Description	iMDI
D0	5	Non-drought	More than 0
D1	4	Near Drought	0.0 to -0.80
D2	3	Moderate drought	-0.80 to -1.10
D3	2	Severe drought	-1.10 to -1.80
D4	1	Extreme drought	-1.40 or less

## 4 Experimental Design, Materials and Methods

### 4.1 Materials

The time-series of MODIS Collection three products (i.e., MOD11A1, MOD09GA, and MOD16A2), were used to calculate environmental indicators. The maximum, average, and minimum values of NDVI, LST, ET, and PET were utilised to calculate drought indices in the study area during the 2001–2020 period. All these images were obtained from the Google Earth Engine platform (https://earthengine.google.com/) and generated at a pixel of 1000 m.

### 4.2 Experimental Design and feature scaling algorithms

Firstly, the vegetation condition index (VCI) was obtained from pixel-based normalization of the NDVI as Eq. (1) [2,12]:

(1) $$VCI=\frac{V_{i,mean}-V_{\min}}{V_{\max}-V_{\min}}$$

where, V*_mean_* is the smoothed pixel values of NDVI; V_max_ and *V*_min_ are maximum NDVI and minimum NDVI, respectively, calculated by the corresponding pixels in same month from the entire NDVI records (2001–2020).

Secondly, the temperature condition index (TCI) is utilized to determine temperature-related vegetation stress [2,13]:

(2) $$TCI_{i}=\frac{T_{\max}-T_{i,mean}}{T_{\max}-T_{\min}}$$

where *T_mean_, T*_max_ and *T*_min_ are the mean, maximum and minimum LST of a period (2001–2020) respectively, and *i* is the year.

Thirdly, evaporative stress index (ESI) involved in evapotranspiration estimation were mainly based on the ratio of ET/PET (ESI, Eq. (3)):

(3) $$ESI_{i}=\frac{ET_{i}}{PET_{i}}$$

Lastly, mathematical functions were used to combine these parameters as an integrated index to come up with the proposed index (iMDI) (Eq. (4)) since their values range from 0 to 1 without any unit. Afterwards, we standardized iMDI as an anomaly to reduce the dissimilarity with missing data of local mean values (Eq. (5)).

(4) $$iMDI_{i}=VCI_{i}+TCI_{i}+ESI_{i}$$

(5) $$Z_{iMDI}=\frac{iMDI_{i}-iMDI_{mean}}{\delta_{iMDI}}$$

where $\delta_{iMDI}$ and $iMDI_{mean}$ are the iMDI's standard deviation and average, respectively. The $Z_{iMDI}$ score is dimensionless and varies in (−∞,+∞) for dryness to wetness conditions, respectively. When there is a drought in a particular year all three indices are low, hence the iMDIi is low. The drought indices are classified as follows: values less than zero indicate drought conditions, while values above zero indicate wet conditions.

### 4.3 Performance assessment

To assess the performance of iMDI, Spearman correlation analyses were conducted to test the correlation between iMDI and the annual field-derived Standardized Precipitation Index (SPI–12) [14] and Reconnaissance Drought Index (RDI–12) [15,16]. These indices are calculated from data measured from weather stations and have been widely used to evaluate the performance of spectral drought indices Cao et al. [17], Shah and Mishra [18], and Wu et al. [5]. The annual mean air temperature and rainfall data from five weather stations in the Vietnamese Central Highlands from 2001 to 2020 were used to calculate the ground-based drought indices (i.e., SPI and RDI). A buffered polygon with an area of one square kilometre at each meteorological station was created to respond the spatial resolution of the MODIS drought index. Afterward, a zone statistic as table of ArcGIS toolbox was applied to perform the mean value of the iMDI at each zone as responding to the weather stations-based drought indices values (i.e., SPI–12 and RDI–12). Finally, an attribute relationship among the weather stations-based drought indices and remotely sensed drought indices was employed at five weather stations with a significance of 95%.

## Ethics Statement

The authors declare that all ethical practices have been followed in relation to the development, writing, and publication of this article.

## CRediT authorship contribution statement

**Thuong V. Tran:** Conceptualization, Formal analysis, Methodology, Data curation, Visualization, Writing – original draft, Writing – review & editing. **Duy X. Tran:** Conceptualization, Formal analysis, Methodology, Writing – review & editing. **Duy B. Nguyen:** Methodology, Writing – review & editing.

## Declaration of Competing Interest

The authors declare that they have no known competing financial interests or personal relationships that could have appeared to influence the work reported in this paper.

## Data Availability

Agricultural Drought in the Vietnamese Central Highlands at 1-km Resolution: Monthly and Annual Datasets (Original data) (Mendeley Data).

## References

[1] Tran T.V., Bruce D., Huang C.-Y., Tran D.X., Myint S.W., Nguyen D.B. (2023). Decadal assessment of agricultural drought in the context of land use land cover change using MODIS multivariate spectral index time-series data. GIScience Remote Sens..

[2] Kogan F.N. (1995). Application of vegetation index and brightness temperature for drought detection. Adv. Space Res..

[3] Anderson M.C., Hain C., Wardlow B., Pimstein A., Mecikalski J.R., Kustas W.P. (2011). Evaluation of drought indices based on thermal remote sensing of evapotranspiration over the continental United States. J. Climate..

[4] West H., Quinn N., Horswell M. (2019). Remote sensing for drought monitoring & impact assessment: progress, past challenges and future opportunities. Remote Sens. Environ..

[5] Wu B., Ma Z., Yan N. (2020). Agricultural drought mitigating indices derived from the changes in drought characteristics. Remote Sens. Environ..

[6] Son B., Park S., Im J., Park S., Ke Y., Quackenbush L.J. (2021). A new drought monitoring approach: vector projection analysis (VPA). Remote Sens. Environ..

[7] UNDRR (2021). United Nations Office for Disaster Risk Reduction.

[8] GSO, General Statistics Office of Vietnam (2021). https://www.gso.gov.vn/en/data-and-statistics/2021/07/statistical-yearbook-of-2020/.

[9] Dinh N.T., Ha N.T.T., Thao N.T.P., Linh N.T. (2017). Geo-Spatial Technologies and Earth Resources.

[10] Nguyen Q.T.N., Nguyen L.D., Nguyen N.D., Nguyen T., Bui L.T., Nguyen L.K. (2017). Phân vùng hạn hán dựa trên chỉ số hạn và mô phỏng chế độ thủy văn trên lưu vực Srepok vùng Tây Nguyên. VNU J. Sci. Earth Environ. Sci..

[11] Nguyen H.T.T., Mai N.T., Bui C.D., Nguyen T.T.P. (2016). Mapping droughts over the central highland of Vietnam in El Niño years using landsat imageries. VNU J. Sci. Soc. Sci. Humanities.

[12] Elhag K., Zhang W. (2018). Monitoring and assessment of drought focused on its impact on sorghum yield over Sudan by using meteorological drought indices for the period 2001–2011. Remote Sens..

[13] Kogan F., Sullivan J. (1993). Development of global drought-watch system using NOAA/AVHRR data. Adv. Space Res..

[14] McKee T.B., Doesken N.J., Kleist J. (1993). Proceedings of the 8th Conference on Applied Climatology.

[15] Tsakiris G., Pangalou D., Vangelis H. (2007). Regional drought assessment based on the reconnaissance drought index (RDI). Water Resour. Manage..

[16] Tsakiris G., Vangelis H. (2005). Establishing a drought index incorporating evapotranspiration. Eur. Water.

[17] Cao Y., Chen S., Wang L., Zhu B., Lu T., Yu Y. (2019). An agricultural drought index for assessing droughts using a water balance method: a case study in Jilin province, Northeast China. Remote Sens..

[18] Shah D., Mishra V. (2020). Integrated drought index (IDI) for drought monitoring and assessment in India. Water Resources Res..

